# Ulcerative Colitis and Crohn’s Disease Are Associated with Decreased Serum Selenium Concentrations and Increased Cardiovascular Risk

**DOI:** 10.3390/nu8120780

**Published:** 2016-12-01

**Authors:** Teresa Castro Aguilar-Tablada, Miguel Navarro-Alarcón, Javier Quesada Granados, Cristina Samaniego Sánchez, José Ángel Rufián-Henares, Flor Nogueras-Lopez

**Affiliations:** 1 Digestive Department, Hospital of Jerez de la Frontera, Cádiz E-11403, Spain; ttablada@hotmail.com; 2 Department of Nutrition and Food Chemistry, Faculty of Pharmacy, University of Granada, Granada E-18071, Spain; quesadag@ugr.es (J.Q.G.); csama@ugr.es (C.S.S.); jarufian@ugr.es (J.Á.R.-H.); 3 Instituto de Investigación Biosanitaria (IBS), University of Granada, Granada E-18012, Spain; 4 Hepatology Department, Granada Hospital Complex, Granada E-18012, Spain; flornogueras@gmail.com

**Keywords:** ulcerative colitis, Crohn’s disease, selenium, influencing factors, nutritional and biochemical markers

## Abstract

The incidence of inflammatory bowel disease (IBD) and associated oxidative stress is increasing. The antioxidant mineral selenium (Se) was measured in serum samples from 106 IBD patients (53 with ulcerative colitis (UC) and 53 with Crohn’s disease (CD)) and from 30 healthy controls. Serum Se concentrations were significantly lower in UC and CD patients than in healthy controls (*p* < 0.001) and significantly lower in CD patients than in UC patients (*p* = 0.006). Se concentrations in patients were significantly influenced by sex, body mass index (BMI), the inflammatory biomarker α-1-antitrypsin, surgery, medical treatment, the severity, extent, and form of the disease and the length of time since onset (*p* < 0.05). Se concentrations in IBD patients were positively and linearly correlated with nutritional (protein, albumin, prealbumin, cholinesterase and total cholesterol) and iron status-related (hemoglobin, Fe and hematocrit) parameters (*p* < 0.05). A greater impairment of serum Se and cardiovascular status was observed in CD than in UC patients. An adequate nutritional Se status is important in IBD patients to minimize the cardiovascular risk associated with increased inflammation biomarkers, especially in undernourished CD patients, and is also related to an improved nutritional and body iron status.

## 1. Introduction

Inflammatory bowel diseases (IBDs) include chronic diseases that affect the gastrointestinal tract, especially the gut [1]. IBDs, which have a significant genetic component, are divided between Crohn's disease (CD) and ulcerative colitis (UC) according to the site involved and the symptoms. Their increased incidence appears to be associated with nutritional imbalances and environmental factors, while an abnormal, exaggerated, and sustained inflammatory response can result from microbiological infections induced by gut microbiota [2,3], with an increase in inflammatory markers [4]. IBDs also produce significant changes in neuronal functions that regulate the bowel, increasing intestinal tract malfunction and the mortality rate [5].

Nutrigenomic research into the effects of food and food components on nutrient-gene interactions has addressed the epigenetic changes responsible for the IBD phenotype [1,6]. Importantly, IBDs have been associated with an increased risk of colon cancer [1,7]. However, despite evidence on the importance of Se and selenoproteins for the cardiovascular (CV) system due to their antioxidant functions [8,9,10] and the role of Se-enriched diets in reducing colon cancer risk development [11], few data are available on the influence of Se in IBDs. Se supplementation has been reported to protect against tissue damage in chemically-induced UC by modifying the expression of genes involved in the mitochondrial regulation of cell death [12]. In addition, a combination of Se and vitamin E was found to exert a protective effect in rats by increasing their overall antioxidant capacity and total thiol levels in the colon [13]. Epidemiological studies have reported reduced Se concentrations in patients with CD or UC [14,15], and clinical studies have evidenced lower blood Se concentrations in IBD patients [5,16,17,18,19]. Se may have a beneficial effect in IBDs by modulating gut inflammation in combination with the gut microbiota [15]. Thus, an increase in selenoproteins deactivates the prostaglandin E_2_ (PGE_2_) secreted by macrophages [14] by changing macrophage polarization from the macrophage-1 (M1) to the macrophage-2 (M2) phenotype, which in turn decreases gut inflammation [20]. These results suggest that higher Se concentrations than are currently recommended may be beneficial for IBD patients.

Our study hypotheses were as follows: (i) Se serum concentrations may be a biomarker of the total Se status of IBD patients [21] and may therefore assist in the differentiation between CD and UC; and (ii) serum Se concentrations in IBD patients are influenced by their body mass index (BMI), length of time with IBD, surgical and/or medical treatment, and the severity, extent, and form of the disease. In order to test these hypotheses, the objectives of this study were: to determine serum Se in patients with IBD (UC and CD patients) and healthy controls from Granada province (SE Spain) using hydride generation atomic absorption spectrometry (HG-AAS); to examine the influence of the aforementioned factors; and to analyze the relationship between IBD and various biochemical and nutritional biomarkers, including those related to inflammatory processes and oxidative stress.

## 2. Experimental Section

### 2.1. Patients and Healthy Controls

The study was carried out in a group of 106 IBD patients from a third-level general hospital in Granada (Southeast Spain): 53 with UC and 53 with CD. The diagnosis and severity of the disease were established by a single gastroenterology consultant (T.C.A.-T) based on the symptoms and results of blood analysis and colonoscopy. Data were gathered on: sex, BMI, length of time with IBD, surgical and/or medical treatment of the patient and the severity, extent, and form (inflammatory, fistulizing, mixed, obstructive, chronic intermittent, or chronic continuous) of the disease. The control group comprised 30 healthy blood donors from the same area. Informed written consent was obtained from all participants for participation in the study, which was conducted in accordance with the Helsinki Declaration. The release of human serum samples was approved by the Ethics Committee of the hospital (Ethic approval code: GHC-970708).

### 2.2. Blood Samples

Blood samples were drawn from the antecubital vein of all participants after overnight fasting and were immediately analyzed by the clinical analysis laboratory of the hospital, using a Hitachi 717 automated analyzer (Englewood, NJ, USA) to determine 26 biochemical and nutritional indexes. Part of the blood sample was left to spontaneously coagulate and was then centrifuged at 3000× *g* for 10 min to obtain the serum, which was frozen and kept at −25 °C until Se determination (see below) at the laboratory of the Nutrition and Food Chemistry Department of the University of Granada.

### 2.3. Total Selenium Measurement

Before the Se determination, serum samples (200 μL) were thawed and homogenized. Sample mineralization was performed using a HNO_3_/HClO_4_ mixture heated at 110 °C in a thermostatic block following a previously optimized procedure [22,23]. After reducing Se (VI) to Se (IV) in HCl, total Se was measured by HG-AAS using a Perkin-Elmer model 1100B atomic absorption spectrometer equipped with Perkin-Elmer MHS-10 hydride generator (Perkin-Elmer, Norwalk, CT, USA). The absorbance in each sample (peak height mode) was correlated with its Se concentration by the addition-calibration method. Reference material 0148 from Contox trace metal serum control Panel C (Kaulson Laboratories Inc., West Caldwel, NJ, USA) was used to establish the accuracy (99.83%) and precision (6.55%) of the method, finding no significant difference (*p* > 0.05) between the concentration obtained (156.4 ± 11.0 μg/L) and the certified concentration (150.5 ± 4.9 μg/L).

### 2.4. Data Analysis

Serum Se values were expressed as means ± standard error of the mean (SEM) and then analyzed by one-way analysis of variance (ANOVA) to evaluate the influence of the study factors listed above; the Student’s *t*-test was used when parametric conditions were met and the Kruskall-Wallis test when they were not. Regression analysis of the relationship of serum Se concentrations with biochemical and nutritional results were performed using Pearson’s test when parametric conditions were met and Spearman’s test when they were not. SPSS 17.0 for Windows (SPSS, Inc., Chicago, IL, USA) was used for the statistical analyses; *p* < 0.05 was considered significant.

## 3. Results

**Figure 1:**
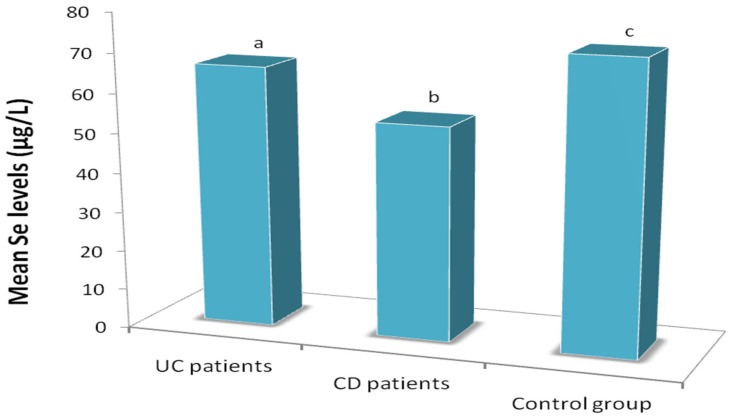
Mean serum Se levels in patients with ulcerative colitis (UC) or Crohn’s disease (CD) and healthy controls (*p* < 0.01; mean values with different letters (a,b,c), significant difference).

Mean serum Se concentrations were significantly lower (*p* = 0.002) in IBD patients than in healthy controls (Figure 1) and were significantly lower (*p* < 0.001) in the patients with CD than in those with UC (Figure 1). Among IBD patients overall and among UC patients, Se concentrations were significantly lower in those who underwent surgery than in those who did not (Table 1).

Serum Se concentrations were significantly influenced (*p* < 0.05) by the severity of UC (Table 2), being significantly higher in those with mild versus severe UC. No significant differences were found for the group with moderate UC, likely due to the small number of these patients (*n* = 4). Among the CD patients, no significant difference was found as a function of disease severity.

Table 3 displays the mean biochemical and nutritional index values obtained in the UC and DC patients. Significant differences (*p* < 0.05) between the UC and CD patients were found in 8 of the 26 biochemical and nutritional indexes studied: γ-globulin, cholesterol, orosomucoid, α-1-antitrypsin, polymorphonuclear count, lymphocyte count, potassium, and C-reactive protein (CRP). Mean levels of inflammatory biomarkers (CRP, orosomucoid and α-1-antitrypsin) were significantly higher and potassium levels significantly lower in CD patients than in UC patients. Greater disease severity was significantly associated with serum α-1-antitrypsin, α-1-globulin, α-2-globulin, orosomucoid and CRP levels in both UC (Table 4) and CD (Table 5) patients.

With respect to the extent and progression of the disease, mean serum Se concentrations were significantly higher in patients with proctosigmoiditis than in those with ileal or colonic involvement (*p* = 0.047; Table 6) and were significantly lower in IBD patients with an inflammatory versus chronic intermittent or chronic continuous form of the disease (*p* < 0.05; Table 7).

As shown in Table 6, no significant differences in serum Se concentrations were observed as a function of the disease extent, categorized according to the Montreal classification [24], in either the UC group (categories: E1 = proctosigmoiditis, E2 = left colitis, and E3 = pancolitis) or the CD group (L1 = ileal; L2 = colic; L3 = Ileocolic; L4 = oral-esophagogastric). In addition, no difference in serum Se concentrations was observed in the CD group (Table 7) as a function of the form of the disease (Montreal classification: B1 = inflammatory; B2 = obstructive; B3 = fistulizing).

Se concentrations were also associated with medical treatment (Table 8) and were significantly lower (*p* < 0.05) in patients treated with 5-acetyl salicylic acid (5-ASA) + corticoids + immunosuppressants (treatment-4); there was also a borderline significant tendency (*p* < 0.07) for Se concentrations to be lower in patients receiving treatment-1 (5-ASA + corticoids) or treatment-3 (corticoids + immunosuppressants).

Serum Se concentrations were significantly lower (*p* < 0.05) in IBD patients with BMI ≤ 18.5 (under-nutrition) than in those with BMI ≥ 30 (obesity) (Table 9). BMI values were positively and linearly correlated with Se concentrations in CD patients (*p* < 0.05; Table 10). Serum Se concentrations in IBD patients were positively correlated with serum protein, albumin, prealbumin, hemoglobin, hematocrit, iron (Fe), β-globulin, and total cholesterol and cholinesterase levels and were negatively correlated with serum α-1 antitrypsin and VSG-2. Finally, Se concentrations were also positively correlated with the length of time of the disease in UC patients and in all IBD patients (Table 10).

With regard to the sex, mean serum Se concentrations were significantly lower in female (53.02 ± 15.33 μg/L) than in male (62.86 ± 21.83 μg/L) IBD patients (*p* < 0.05).

## 4. Discussion

In this study, mean serum Se concentrations were lower in IBD patients than in healthy controls, as previously reported by other researchers [5,16,17,18,19]. One study [25] found a significant difference between patients with pediatric-onset IBD and healthy controls, likely related to differences in Se concentrations and disease development in this age group; thus, serum Se concentrations were considerably higher than in the present study population. Mean serum Se concentrations were also higher in patients with UC than in those with CD in the present series. Some inflammatory biomarkers (e.g., CRP, orosomucoid and α-1 antitrypsin) were higher in CD than UC patients, and micronutrient deficiencies were more frequent in CD than UC patients, in agreement with previous findings [26]. Alongside the greater alteration of biochemical and nutritional biomarkers (K and total cholesterol levels) found in CD patients, these results indicate a more compromised nutritional and inflammation status, with increased oxidative stress and CV risk in patients with CD than in those with UC.

Surgery in UC patients involves colectomy, which would be responsible for their lower Se concentrations rather than the disease itself, whereas CD patients are treated with partial resection of the small intestine and a colectomy is not usually necessary, which would explain the lack of a decrease in their serum Se concentrations.

Serum Se concentrations were significantly influenced by the severity of UC, being higher in those with mild versus severe UC. However, no IBD patient with inactive UC was available during the sampling period, which is a study limitation.

The inflammatory biomarkers RCP, orosomucoid and α-1 antitrypsin were increased in the IBD patients. Thus, α-1 antitrypsin was inversely and linearly correlated with serum Se concentrations in all IBD patients and in the UC patients, suggesting that the enhanced inflammatory status (higher α-1 antitrypsin levels) of IBD patients may produce a marked decrease in serum Se concentrations. Inflammatory biomarker values were higher with greater disease severity and with lower serum Se concentrations and these differences were more marked in IBD patients who had undergone surgery. Hence, the increased inflammatory status of IBD patients may give rise to an increase in oxidative stress and in CV risk. We highlight the lower serum Se concentrations in patients with the inflammatory form of IBD than in those with chronic intermittent or chronic continuous forms, supporting the key role of the higher inflammatory status of IBD patients in their reduced Se concentrations.

A striking and novel finding was the positive and linear correlation of serum Se with iron status-related biochemical biomarkers (hemoglobin, serum Fe and hematocrit) in both CD and UC patients and with β-globulin in UC patients. Hence, achievement of an appropriate body Se status in CD and UC patients is important to maintain an adequate iron status in both groups of patients. The combination of a low body Fe status and reduced serum Se concentration is frequently observed in IBD patients. A positive association was found in the present patients between serum Se concentrations and biochemical parameters related to Fe status. One explanation for this association may be that animal protein foods, often recommended to IBD patients, are a good dietary source of both bioavailable Se and bioavailable Fe [8].

Serum Se concentrations were lower in IBD patients treated with 5-ASA + corticoids + immunosuppressants and tended to be lower in those treated with 5-ASA + corticoids or with corticoids + immunosuppressants in comparison to non-treated patients. These findings may point to a possible relationship between corticoids and a greater reduction in serum Se concentrations in IBD patients. There is a need to verify this proposition in future studies that include IBD patients receiving corticoids alone. It should also be taken into account that 5-ASA + immunosuppressant treatment is recommended for the most aggressive and active forms of IBD until the disease is resolved, and they are combined with corticosteroids to treat patients with advanced IBD and high inflammation. Hence, the reduced serum Se concentrations in the IBD patients treated with 5-ASA + corticoids + immunosuppressants may be related to the higher severity in patients receiving this treatment as well as to their receipt of corticosteroids.

Serum Se concentrations were lower in patients with BMI < 18.5 kg/m^2^ (undernourishment) than in those with BMI ≥ 30 (obesity). Therefore, an improved nutritional status implies an improved body Se status in IBD patients. Serum Se concentrations were also increased in patients with a longer history of the disease, which may be attributable to the effects of their medical treatment.

## 5. Conclusions

Serum Se concentrations were significantly lower in IBD patients than in healthy controls and were significantly lower in patients with Crohn’s disease (CD) than in those with ulcerative colitis (UC). Among UC patients, serum levels were lower in those who had undergone a colectomy than in those who had not. The greater increase in various inflammatory biomarkers in CD patients may be associated with their higher oxidative stress and cardiovascular risk in comparison to UC patients. In UC patients, worse disease severity was correlated with a greater reduction in Se concentrations. Among these IBD patients, serum Se concentrations were lower in those who were undernourished, in those treated with 5-ASA + corticoids + immunosuppressants, and in those with the inflammatory form of the disease. Finally, higher serum Se concentrations were associated with improved nutritional status and iron status biomarkers in IBD patients, likely related to their recommended animal protein-rich diet, which supplies bioavailable Se and Fe. Serum Se concentrations were also increased in patients with a longer history of the disease, which may be attributable to the effects of their medical treatment.

## Figures and Tables

**Table 1:** Serum Se concentrations of IBD patients as a function of surgery (yes/no).

Surgery	UC	CD	All IBD Patients
	(Mean ± SEM; µg/L)	(Mean ± SEM; µg/L)	(Mean ± SEM; µg/L)
Yes	41.13 ± 15.91 ^a^	52.3 ± 3.55	51.3 ± 3.58 ^a^
No	64.7 ± 2.74 ^b^	55.3 ± 3.58	61.0 ± 2.23 ^b^

^a,b^ different superscripts in same column, significant difference (*p* < 0.05); IBD, Inflammatory Bowel Disease; UC, Ulcerative Colitis; CD, Crohn’s Disease.

**Table 2:** Mean selenium concentrations of IBD patients as a function of disease severity.

IBD Patients	*n*	Mean Se ± SEM (μg/L)	Range (μg/L)
UC patients			
Mild	32	68.8 ± 2.91 ^a^	45.5–111.8
Moderate	4	55.2 ± 7.38 ^a,b^	41.3–66.5
Severe	17	49.3 ± 5.72 ^b^	12.4–91.1
CD patients			
Inactive	17	56.5 ± 3.71	37.2–99.3
Mild	21	54.4 ± 3.79	20.6–86.9
Moderate	9	50.6 ± 8.07	12.4–103.5
Severe	6	44.8 ± 6.81	24.8–62.1

^a,b^ different superscripts in same column, significant difference (*p* < 0.05).

**Table 3:** Biochemical values showing significant difference (*p* < 0.05) between UC and CD patients.

Biochemical Parameters	UC (Mean ± SEM; µg/L)	CD (Mean ± SEM; µg/L)	*p* ^a^
γ-globulin (g/dL)	1.35 ± 0.07	1.14 ± 0.04	0.011
Total Cholesterol (mg/dL)	194 ± 6.45	165 ± 5.24	0.001
Orosomucoid (mg/dL)	117 ± 6.46	139 ± 9.03	0.055
α-1-antitrypsin (mg/dL)	192 ± 8.38	227 ± 8.60	0.005
Polymorphonuclear count (%)	62.0 ± 1.32	67.4 ± 1.33	0.005
Lymphocyte count (%)	28.3 ± 1.22	22.1 ± 1.17	0.000
K (mEq/L)	4.35 ± 0.06	4.06 ± 0.06	0.001
RCP (mEq/L)	1.42 ± 0.43	3.42 ± 0.73	0.019

^a^ *p*, level of significance.

**Table 4:** Plasma inflammatory biomarkers in UC Patients as a function of severity.

Biochemical Parameter	Mild CU	Moderate CU	Severe CU	*p* ^a^
	(Mean ± SEM)	(Mean ± SEM)	(Mean ± SEM)	
α-1-antitrypsin (mg/dL)	179.5 ± 8.04	220.3 ± 17.35	290.0 ± 59.24	0.031
α-2-globulin (g/L)	0.641 ± 0.033	0.722 ± 0.053	0.943 ± 0.140	0.035
Orosomucoid (mg/dL)	98.04 ± 5.71	146.3 ± 11.68	207.7 ± 40.41	<0.001
α-1-globulin (g/L)	0.431 ± 0.020	0.524 ± 0.037	0.713 ± 0.060	0.014
CRP (mEq/L)	0.610 ± 0.137	2.287 ± 1.014	8.100 ± 4.750	0.003

^a^ *p*, level of significance.

**Table 5:** Plasma inflammatory biomarker levels of CD patients as a function of severity.

Biochemical Parameter	Inactive EC	Mild EC	Moderate EC	Severe EC	*p* ^a^
	(Mean ± SEM)	(Mean ± SEM)	(Mean ± SEM)	(Mean ± SEM)	
α-1-antitrypsin (mg/dL)	201.8 ± 10.80	209.0 ± 8.31	262.1 ± 19.08	318.0 ± 15.59	<0.001
α-2-globulin (g/L)	0.624 ± 0.019	0.654 ± 0.027	0.869 ± 0.055	0.968 ± 0.107	<0.001
Orosomucoid (md/dL)	92.97 ± 4.039	115.2 ± 6.993	183.3 ± 24.39	258.1 ± 11.23	<0.001
α-1-globulin (g/L)	0.412 ± 0.023	0.470 ± 0.016	0.606 ± 0.037	0.718 ± 0.051	0.009
CRP (mEq/L)	0.769 ± 0.192	1.564 ± 0.613	6.922 ± 2.360	12.18 ± 2.371	<0.001

^a^ *p*, level of significance.

**Table 6:** Serum Se concentrations of IBD patients as a function of disease extent.

Disease Extent	*n*	Mean ± SEM (μg/L)	Range (μg/L)
Proctosigmoiditis EC	15	70.1 ± 4.67 ^a^	49.6–111.8
Left colitis EC	17	62.1 ± 4.50 ^a,b,c^	41.3–103.5
Pancolitis EC	21	59.6 ± 4.70 ^a,b,c^	12.4–99.3
Ileal CD	18	52.4 ± 5.76 ^c.b^	12.4–103.5
Ileocolic CD	15	60.4 ± 4.93 ^a,b,c^	28.9–99.3
Colic CD	15	55.0 ± 2.36 ^b,c^	41.3–74.5
Oral-esophagogastric CD	3	38.6 ± 8.40 ^a,b,c^	24.8–53.8
Enteritis CD	2	43.4 ± 6.22 ^a,b,c^	37.2–50.0

^a,b,c^ different superscripts, significant difference (*p* = 0.047).

**Table 7:** Serum Se concentrations in IBD patients as a function of the form of the disease.

Form of Disease	*n*	Mean ± SEM (μg/L)	Range (μg/L)
Chronic Intermittent UC	40	64.0 ± 3.48 ^b^	12.4–111.8
Chronic Continuous UC	13	62.1 ± 3.76 ^b^	49.6–91.15
Obstructive CD	10	55.4 ± 7.79 ^a,b^	20.6–103.5
Fistulizing CD	8	57.4 ± 8.86 ^a,b^	12.4–99.3
Inflammatory CD	21	52.0 ± 2.66 ^a^	24.8–86.9
Mixed CD	14	54.7 ± 5.42 ^a,b^	28.9–99.3

^a,b^ different superscript, significant difference (*p* = 0.082). Multiple range test results show significantly lower serum Se concentrations in the inflammatory form than in the chronic intermittent or chronic continuous forms.

**Table 8:** Serum Se Concentrations of IBD patients as a function of medical treatment.

Medical Treatment	Treated IBD Patients	Non-Treated IBD Patients	*p* ^a^
	(Se, µg/L: Mean ± SEM)	(Se, µg/L: Mean ± SEM)	
Treatment-1 (5-ASA + Corticoids)	55.0 ± 1.99	64.1 ± 3.57	0.057
Treatment-2 (5-ASA + Immunosuppressants)	56.3 ± 2.95	60.0 ± 2.14	0.112
Treatment-3 (Corticoids + Immunosuppressants)	54.5 ± 2.75	60.1 ± 2.36	0.070
Treatment-4 (5-ASA + Corticoids + Immunosuppressants)	52.1 ± 2.95	60.6 ± 2.35	0.018

^a^ *p*, level of significance.

**Table 9:** Serum Se of IBD patients as a function of body mass index.

BMI	*n*	Mean ± SEM (μg/L)	Range (μg/L)
≤18.5 (Undernutrition)	15	42.7 ± 2.77 ^a^	37.2–45.5
18.5–24.9 (Normal Weight)	40	61.2 ± 3.14	24.8–99.3
25–29.9 (Overweight)	36	61.7 ± 3.58	20.6–111.8
≥30 (Obesity)	15	65.8 ± 4.39 ^b^	45.5–91.1

^a,b^ different superscript, significant difference (*p* = 0.019).

**Table 10:** Correlation analysis results showing significant and borderline significant differences in serum Se concentrations as a function of biochemical index/time with disease.

Biochemical Index/Time with Disease	UC	CD	All IBD Patients
Protein	0.358 (0.000)	0.320 (0.005)	0.423 (0.000)
Albumin	0.466 (0.000)	0.447 (0.001)	0.429 (0.000)
Prealbumin	0.349 (0.011)	0.366 (0.011)	0.376 (0.000)
Hemoglobin	0.554 (0.000)	0.350 (0.011)	0.481 (0.000)
Hematocrit	0.579 (0.000)	0.330 (0.005)	0.497 (0.000)
Fe	0.272 (0.047)	0.454 (0.001)	0.385 (0.001)
β-globulin	0.454 (0.001)	-	0.223 (0.023)
Total Cholesterol	0.353 (0.009)	0.265 (0.060)	0.355 (0.000)
Cholinesterase	0.495 (0.000)	0.404 (0.005)	0.418 (0.000)
α-1-antitrypsin	−0.269 (0.052)	-	−0.257 (0.009)
VSG-2	-	−0.268 (0.063)	−0.264 (0.008)
BMI	-	0.294 (0.059)	0.223 (0.023)
Time with disease	0.353 (0.009)	-	0.301 (0.002)
